# Determinants of the number of mammography units in 31 countries with significant mammography screening

**DOI:** 10.1038/sj.bjc.6604657

**Published:** 2008-09-09

**Authors:** P Autier, D A Ouakrim

**Affiliations:** 1 Epidemiology Methods and Support Group, International Agency for Research on Cancer, Lyon, France

**Keywords:** breast cancer, mammography screening, radiology

## Abstract

In the 2000s, most of the female population of industrialised countries had access to mammography breast cancer screening, but with variable modalities among the countries. We assessed the number of mammography units (MUs) in 31 European, North American and Asian countries where significant mammography activity has existed for over 10 years, collecting data on the number of such units and of radiologists by contacting institutions in each country likely to provide the relevant information. Around 2004, there were 32 324 MU in 31 countries, the number per million women ranging from less than 25 in Turkey, Denmark, the Netherlands, the United Kingdom, Norway, Poland and Hungary to more than 80 in Cyprus, Italy, France, the United States and Austria. In a multivariate analysis, the number of MUs was positively associated with the number of radiologists (*P*=0.0081), the number of women (*P*=0.0023) and somewhat with the country surface area (*P*=0.077). There is considerable variation in the density of MU across countries and the number of MUs in service are often well above what would be necessary according to local screening recommendations. High number of MUs in some countries may have undesirable consequences, such as unnecessarily high screening frequency and decreased age at which screening is started.

Biennial mammography screening is considered to reduce breast cancer mortality by 25% in women aged 50–69 years (IARC, 2002). In women aged 40–49 years, annual screening seems to reduce breast cancer mortality by 15–17% (Moss *et al*, 2006). Since the beginning of this millennium, most women living in industrialised nations have had access to mammography screening. Therefore, for instance, in 2005, ⩾70% of women aged 50–69 years participated in mammography screening in the Netherlands, France, Norway, the United Kingdom and the United States (OECD, 2007). However, there is considerable variation among countries (and sometimes also between counties or provinces) in mammography screening, including the age groups that are recommended for screening and those for which it is reimbursed by health insurance, and in the frequency of mammography (IARC, 2002; Lynge *et al*, 2003; Smith-Bindman *et al*, 2003; Yankaskas *et al*, 2004; USPSTF, 2008). Attendance can be by invitation from a screening programme, self-reference, a doctor's referral or through a combination of these three. Variation in all these factors may influence the number of mammography units (MUs) in countries. The objective of this study was to estimate the number of MUs in European, North American and Asian countries where significant mammography screening activity has existed for over 10 years.

## Materials and methods

For 34 countries, using address lists obtained from the International Agency for Research on Cancer (IARC) and through internet searches, we gathered a list of potential sources of information. For some countries, the data were readily available in published reports or on websites; it was nonetheless verified through direct contact with the sources. We wrote to all potential sources of information we identified, asking for information on (i) the total number of MUs (analogic and digital) and (ii) the total number of radiologists, with numbers specialising in mammography.

The letter clearly stated that data sent to the IARC would be used to make a comparison among countries. If a contact could not provide relevant data, he or she was asked to provide us the details of an appropriate institution or to forward our letter directly to this institution.

We considered an MU to be any X-ray machine used for breast examination, through either analogical or digital modalities. As the same equipment could serve for both diagnosis and screening purposes, we made no distinction between MU declared as serving these purposes or reported as being part of a national screening programme or a medical facility (e.g., hospital, breast clinic, private radiology practice).

Between March and December 2006, we had contact with 229 potential sources of data, many of which forwarded our request to more appropriate data source (details can be obtained from the authors). We received data from 123 institutions or companies. When we obtained data from several sources for one country, we gave priority to radioprotection institutes, as registration of X-ray-emitting devises is compulsory in all countries. Sometimes, however, governmental radioprotection offices are established at a sub-national rather than at a national level precluding the identification of any single body having the relevant information for the entire country. When radioprotection institutes did not answer, or were not available at a national level, we turned to alternative sources of information. When several sources responded, we used the one most likely to be aware of MU in the country. Information from social security offices was usually not considered, as for these institutions, a clinic or a radiological facility is usually considered as a single ‘mammography centre’ although it may comprise more than one mammography unit. When dissimilar data from at least two *a priori* reliable sources were received for a country, we verified the information by re-sending the letter to these sources and, when possible, to other contacts. If for a country, no source of MU data was found, we used data from the European Coordination Committee of the Radiological and Electronical Industry (COCIR, 2003) or from the Organisation for Economic Cooperation and Development (OECD, 2007). If the number of radiologists in a country could not be obtained, we used data from the European Association of Radiology (EAR, 2005). Data selected for each country are listed in Table 1.

As only five countries (Finland, Ireland, the Netherlands, Sweden and the United Kingdom) provided separate counts of MU used in national screening programmes and in other medical facilities, we did not use these in our analysis. Some countries gave data on digital MU; given the rapid changes in digital mammography equipment during the 2000s, it was considered premature to provide these statistics.

We collected information of country breast screening practice through literature search (e.g., Lynge *et al*, 2003; Yankaskas *et al*, 2004) and information gathered at the IARC. This information was not requested to institutions contacted for the number of MUs, as it was often known to be unavailable.

For each country, we computed the number of MUs divided by the number of women in 2005. The population data source was the Population Division of the Department of Economic and Social Affairs of the United Nations (ESA, 2007). For defining the number of MUs that would be necessary in a country, we took as a basis the Netherlands and the United Kingdom, two countries with national mammography screening programmes, where screening outside the national programme is rare and where a participation of at least 70% of the population was reached in 1995 in the United Kingdom (women aged 50–64 years, triennial screening) (ACBCS, 2006) and in 1997 in the Netherlands (women aged 50–69 years, biennial screening) (Otto *et al*, 2003). Computations in Table 2 are based on data from the Netherlands because triennial screening schedule exists only in the United Kingdom. We assumed three sets of recommendations: (i) biennial screening of women 50–69 years old, (ii) annual screening for women aged 40–49 years and of biennial screening at 50–69 years and (iii) annual screening at 40–69 years. The last scenario corresponds to recommendations made in the United States by the American Medical Association, the American College of Radiology and the American Cancer Society (USPSTF, 2008). In the first, second and third scenarios, about 20, 46 and 66 MU per million women would be necessary, respectively.

Using least square linear regression, we fitted a multivariate model for the prediction of the number of MUs according the to number of women of all ages, of radiologists and of country surface. We fitted another model for European Union Member States to examine the relationship between the number of MUs and the percentage of women who had a mammography in the last 12 months. The latter data were taken from a survey done in the European Union in 2006 that reported the percentages of women 50 years old and over who had a mammography examination in the last 12 months, regardless of whether it was for screening or for diagnostic purposes (Eurobarometer, 2007). The survey distinguished between examination done after receiving an invitation to attend the screening programme and that through woman's own initiative and that through a doctor's initiative.

This study has been approved by the Institutional Review Board of the IARC.

## Results

Of the 34 countries studied, we could not find data on the number of MUs in three and on the number of radiologists in seven countries. Data on the number of MUs were thus available for 31 countries, and data on the number of radiologist were available for 27 countries. Germany was the only country for which we could not obtain data more recent than 2001.

Around 2004, there were 32 324 MU in 31 countries where significant mammography screening was established. The number of MUs per million women ranged from 13 in Turkey to 100 in Austria (Table 3). There were less than 25 MU per million women in Turkey, Denmark, the Netherlands, the United Kingdom, Norway, Poland and Hungary, whereas there were more than 80 in Cyprus, Italy, France, the United States and Austria. Sixteen countries had more than 46 MU per million women, and seven had more than 66 MU per million women.

Acquisition of digital mammography equipments was most noticeable in Austria, Finland, France, Norway, Switzerland, Japan and the United States, but data are not shown as the change from analogical to digital mammography is now taking place rapidly in a number of countries.

Eleven countries reported the number of radiologists specialised in mammography examination (Table 3), ranging from 7% in South Korea to 62% in Canada. In spite of the great variability in the proportion of radiologists reported as being specialised in mammography examination, a positive correlation existed between the total number of radiologists and the number of radiologists specialised in mammography examination (Pearson *r* coefficient=0.80, *P*=0.0024). We then examined how female population size, the number of radiologists and country surface influenced the number of MUs by fitting a linear regression (Table 4). Both female population size and the number of radiologists predicted the number of MUs, whereas country surface was a less good predictor. More complex models, including for instance variables related to age groups being actually screened (when available) or population density, were not better predictors of the number of MUs.

**Figure 1:**
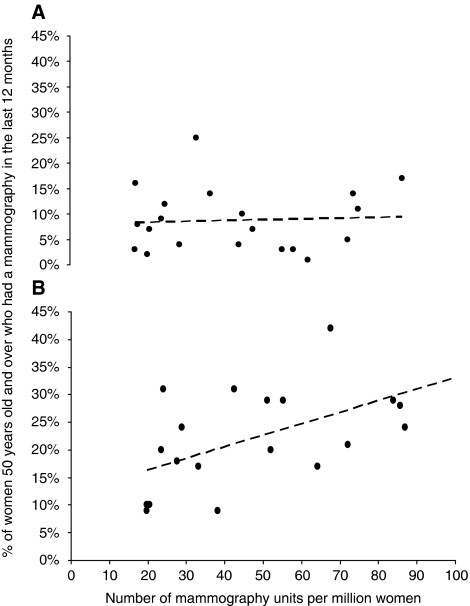
Relationship between the number of mammography units and the percentage of women 50 years old and more in 21 countries of the European Union reporting a mammography done in the last 12 months related to (**A**) an invitation to attend mammography screening (Pearson's *r* coefficient=0.06, *P*=0.82); (**B**) own desire to have a mammography screening or prescribed by a doctor (Pearson's *r* coefficient=0.58, *P*=0.0074). Data on mammography use from Eurobarometer (2007).

In Member States of the European Union, the number of MUs was a good predictor of attendance to mammography screening when attendance was due to self-reference or due to doctor's prescription, but not after invitation by a breast cancer screening programme (Figure 1).

## Discussion

This study shows the considerable variability in density of MU across countries, and the number of MUs in service often exceeds what would be necessary to fulfil local screening recommendations. Country-specific volumes of breast cancer screening activities were not examined because reliable quantitative data were not generally available (Lynge *et al*, 2003; Yankaskas *et al*, 2004). Similarly, age at screening and screening frequency could not be included in regression models. A strong discrepancy often exists between recommendations and actual practice. For instance, in France, biennial screening is recommended for women aged 50–74 years, whereas as many as 60% of French women aged 40–49 years reported at least one recent screening (Spyckerelle *et al*, 2002). Furthermore, recommendations may differ within the same country; according to health organisation in the United States, seven bodies have issued different recommendations on age and frequency of screening (USPSTF, 2008).

The few data we had on the number of radiologists specialised in mammography examinations suggested that the total radiologists registered in a country could represent a reasonable approximation to those specialising in mammography. But the variability in radiologists specialising between countries probably reflects differences in what this entails. In some countries, geographical distances may lead to installation of more MUs for easier access to screening. The multivariate model we fitted showed borderline statistical association between country surface and the number of MUs, once the number of radiologists and of women was taken into account. However, similar densities observed in countries much larger than the Netherlands, Norway and the United Kingdom indicate that geographical factors cannot account for all the difference in density of MU. Hence, all countries considered together, both total female population and the number of radiologists established in the country were the essential determinants of the number of MUs, irrespective of country size.

Our data are more recent than the COCIR report (COCIR, 2003) and cover more countries than the OECD reports (OECD, 2007). Good agreement was found between our data and OECD data, except that Spain, for which the OECD admitted that their data could be underestimated (we received data from the Sociedad Espanola de Diagnostico por Imagen de la Mama, see Table 1), and for Korea, where the OECD got data from the Health Insurance Review Agency, whereas our data came from the Korean Association for Radiation Protection (Table 1), which was probably more reliable than the former.

Examination of MU density in relation to the most recent mortality data (Héry *et al*, 2008a, 2008b) shows no evidence of a correlation. In fact, until the late 1990s, breast cancer mortality remained practically unchanged in some countries with a high MU density (e.g., Belgium, France), whereas it decreased substantially in several countries with a low density of MU (e.g., the United Kingdom, the Netherlands).

Coverage of the female population at ages 50–69 years was not achieved in Turkey and Denmark in 2003, though in Turkey, the number of MU may have been underestimated (Voyvoda *et al*, 2007). In Denmark, in 2003, mammography screening was offered to about 20% of women aged 50–69 years, and there was practically no provision outside the national programme (Jensen *et al*, 2004). A participation of the target population to the screening programme of at least 70% was reached in 1995 in the United Kingdom (women 50–64 years old, triennial screening) (ACBCS, 2006), in 1997 in the Netherlands (women 50–69 years old, biennial screening) (Otto *et al*, 2003), and in 2004 in Norway (women 50–69 years old, biennial screening) (Hofvind *et al*, 2007; Vatten, 2007). The main differences between these three countries and most other countries were the higher screening frequencies and broader age groups to whom screening was offered, by national programmes or by doctors.

Sixteen of the 31 countries included had more than 46 MU per million women and five have about twice this density. These data suggest that in many countries the number of MUs is well above what would be necessary according to local screening recommendations, and oversupply of MU may exist, peaking in France, Cyprus, the United States, Austria and Italy. An oversupply of MU may have undesirable consequences (Brown *et al*, 1990), which are listed below. (i) Insufficient experience of radiologists in the interpretation of mammograms for optimal sensitivity and specificity (Smith-Bindman *et al*, 2005; Théberge *et al*, 2005). (ii) The broadening of age ranges in which mammography is offered, mainly women less than 40 years old. For instance, in Germany, 18% of first mammographies were in women below 30 years and 31% were in women aged 30–39 years (Klug *et al*, 2005). In United States and in France, 47 and 45% respectively of first mammographies were in women below 40 years (Spyckerelle *et al*, 2002; Colbert *et al*, 2004). (iii) An increasing frequency of mammography. (iv) Increased costs of screening because of the necessity to amortise and to pay the running costs of mammography centres.

The enforcement of the Mammography Quality Standard Act in the United States in 1992 did not notably reduce the number of MUs, but probably led to the creation of mammography facilities that could better apply quality assurance requirements (Fischer *et al*, 1998; Destouet *et al*, 2005).

The European Guidelines for Quality Assurance in Breast Cancer Screening and Diagnosis exist since 1993 (Perry *et al*, 2006). There are no data on the likely impact of these guidelines on the installation of MU in European countries. An essential feature of the European guidelines not present in the United States is the recommendation to implement regular invitations to women for mammography screening to maximise participation and regularity. The positive correlation in Europe between the number of MUs per million women and self-referred or prescribed participation in mammography screening (and not after invitation) suggests that globally speaking, screening attendance in the European Union is not related to invitations by the programmes but rather to the offering of mammography screening, which is itself tightly related to the number of radiologists. In this respect, in high MU-density countries, the introduction of an invitation-only programme could not absorb and support the costs of the already functioning mammography services. In such cases, such an introduction would not, therefore, improve participation and reduce avoiding unnecessary screening, including outside the recommended age range.

## Figures and Tables

**Table 1:** Sources of data on numbers of mammography (MM) units and radiologists

			**Information on:**	
**Country**	**Institute or company^a^**	**City**	**No. of radiologists**	**No. of MM units**
Australia	Australian Institute of Radiography	Victoria	X	X
Austria	Austrian Medical Chamber	Vienna	X	
	Austrian Research Centre Seiberdorf	Seiberdorf		X
Belgium	Agence Fédérale de Contrôle Nucléaire	Bruxelles		X
	Royal Belgian Society of Radiology	Bruxelles		X
	SPF Santé Publique, Sécurité de la Chaîne Alimentaire et Environnement	Bruxelles	X	
Canada	Mammography Accreditation Program MAP	Québec	X	X
	The Royal College of Physicians and Surgeons of Canada	Ottawa	X	
Cyprus	Cyprus Medical Device Authority	Pallouriotissa		X
Czech Republic	Charles University in Prague	Prague	X	X
Denmark	European Association of Radiology (EAR, 2005)		X	
	Institute of Radiation Hygiene of Denmark^b^	Copenhagen		X
Estonia	*No Information found*			
Finland	Radiation and Nuclear Safety Authority Radiation Protection	Helsinki		X
France	Agence Française de Securité Sanitaire des Produits de Santé	Paris		X
	Conseil National de l′Ordre des Médecins	Paris	X	
Germany	Coordination Committee of the Radiological and Electronical Industry (COCIR, 2003)			X
	The National Association of Statutory Health Insurance Physicians (KVB)	Berlin	X	
Greece	European Association of Radiology (EAR, 2005)		X	
	Hellenic Ministry of Health^b^	Athens		X
Hungary	Health Physics Section (Roland Eötvös Phys Soc) of Hungary	Budapest	X	
	Hungarian National Institute for Hospital and Medical Engineering^b^			X
	Hungarian Society of Radiologists	Budapest	X	X
Iceland	Iceland Cancer Registry and Iceland Cancer Society	Reykjavik	X	X
Ireland	Breast Check, The National Breast Cancer Screening Program	Dublin		X
	European Association of Radiology (EAR, 2005)		X	
	Radiological Protection Institute of Ireland	Dublin		X
Italy	Società Italiana di Radiologia Medica	Milano	X	X
Japan	Japan Radiological Society	Tokyo		X
	Ministry of Health, Labour and Welfare		X	
South Korea	Korean Association for Radiation Protection	Seoul	X	X
Lithuania	*No information found*			
Luxembourg	Ministère de la Santé	Luxembourg	X	X
Malta	Malta Standards Authority	Valletta		X
New Zealand	National Radiation Laboratory, a division of the Ministry of Health^b^			X
Norway	European Association of Radiology (EAR, 2005)		X	
	Norwegian Breast Cancer Screening Programme	Oslo		X
	The Norwegien Radiation Protection Authority	Oslo		X
Poland	Nofer Institute of Occupational Medicine	Lodz	X	X
	Radiation Protection Section Polish Society of Medical Physics	Warsaw		X
Portugal	Ministry of Health Competent Authority^b^			X
	Ordem dos Médicos	Lisbon	X	
Slovac Republic	Soc of Nucl Med and Rad.Hygiene/Rad.Prot.Section	Bratislava	X	X
Slovenia	*No information found*			
Spain	Sociedad Espanola de Diagnostico por Imagen de la Mama	Madrid	X	X
Sweden	Swedish Medical Association	Stockholm	X	
	Swedish Radiation Protection Authority	Stockholm		X
Switzerland	Office Fédéral de la Santé Publique	Bern	X	X
The Netherlands	Radiological Society of the Netherlands	s-Hertogenbosch	X	X
Turkey	Turkish Atomic Energy Commission adapted by Voyvoda *et al* (2007)			X
United Kingdom	NHS Cancer Screening Programmes	Sheffield	X	X
USA	Food and Drug Administration	Rockville, MD		X
	Medical Marketing Service Inc	Wood Dale, IL	X	

a The complete list of institutions contacted in each country and the 122 institutions or companies that sent data can be obtained from the authors.

b Data obtained from OECD (2007).

**Table 2:** Estimation of number of mammography (MM) units for annual screening of women 40–49 years old and biennial screening of women 50–69 years old, taking number of MM units in the Netherlands

**Computation no.**	**Parameter**	**Computations**	**Results**
(1)	Number of women of all ages in 2005 (million)		8.208
(2)	Number of women 50–69 years old in 2005 (million)		1.881
(3)	Number of women 40–49 years old in 2005 (million)		1.247
(4)	Number of MM units, biennial screening of women 50–69^*^ years old		162^*^
(5)	Number of MM units, if annual screening of women 50–69 years old	(4)^*^2	324
(6)	Number of MM units per million women of all ages, biennial screening of women 50–69 years old	(4)/(1)	20
(7)	Number of MM units per million of women 50–69 years old, biennial screening	(4)/(2)	86
(8)	Number of MM units to install for annual screening of women 40–49 years old	(3)^*^(7)^*^2	215
(9)	Total number of MM units, annual screening of women 40–49 years old, and biennial screening of women 50–69 years old	(4)+(8)	377
(10)	Total number of MM units, annual screening of women 40–69 years old	(5)+(8)	539
(11)	Number of MM units per million women of all ages, annual screening of women 40–49 years old, and biennial screening of women 50–69 years old	(9)/(1)	46
(12)	Number of MM units per million women of all ages, annual screening of women 40–69 years old	(10)/(1)	66

^*^Number of MM units in the Netherlands in 2005.

**Table 3:** Number of radiologists and of mammography units in 31 countries^a^

**Country**	**Number of women of all ages in year 2005^b^**	**Number of radiologists after 2002**	**Number of radiologists reported as specialised in mammography examination**	**Total number of mammography units**	**Mammography units per million women**	**Year of data for mammography units**
Turkey	36 314 381	NA	NA	493	14	2006
Denmark^c^	2 742 913	1050	NA	54	20	2003
The Netherlands^d^	8 208 045	829	171	162	20	2005^e^
United Kingdom^d^	30 514 714	2911	301	626	21	2005
Norway	2 325 518	430	NA	51	22	2006
Poland	19 844 491	2400	300	466	23	2005^e^
Hungary	5 289 951	1200	180	127	24	2004
Czech Republic	5 244 887	1293	NA	145	28	2003
Slovac Repubic	2 780 891	530	118	80	29	2005^e^
Ireland	2 084 588	180	NA	69	33	2005
Iceland	147 000	26	NA	5	34	2007
Sweden	4 554 814	974	NA	174	38	2006
Canada	16 274 553	2039	1,259	656	40	2006
Luxembourg	235 830	58	NA	10	42	2006
New Zealand	2 048 740	268	NA	94	46	2004
Korea	23 844 230	2627	189	1136	48	2005
Japan	65 506 343	10 556	1641	3,207	49	2005^e^
Germany	42 301 156	6314	NA	2,163	51	2001
Spain	21 915 968	3895	371	1,140	52	2004
Belgium	5 306 707	1466	450	293	55	2006
Australia	10 202 449	1334	NA	645	63	2005^e^
Malta	202 454	NA	NA	13	64	2006
Finland	2 679 104	NA	NA	179	67	2006
Portugal	5 422 193	762	NA	366	68	2005
Greece	5 625 709	2500	NA	405	72	2005
Switzerland	3 740 073	654	NA	297	79	2005
Cyprus	428 936	NA	NA	36	84	2006
Italy	29 898 180	10 000	1147	2560	86	2005^e^
France	31 032 618	7392	NA	2700	87	2006
USA	151 532 730	24 913	NA	13 552	89	2006
Austria	4 186 019	950	150	420	100	2005^e^

a Mammography units include analogical and digital machines, being part or not being part of a national screening programme.

b From the Population Division of the Department of Economic and Social Affairs of the United Nations.

c Mammography screening programme organised in Copenhagen city and in two counties, covering 20% of Danish women 50–69 years of age (Jensen *et al*, 2004).

d Coverage of target population of 70% or more was achieved in 1995 in the United Kingdom (women 50–64 years old, triennial screening) (ACBCS, 2006), in 1997 in the Netherlands (women 50–69 years old, biennial screening) (Otto *et al*, 2003) and in 2004 in Norway (women 50–69 years old, biennial screening)(Vatten, 2007; Hofvind *et al*, 2007).

e Year of inventory not specified by data source and assumed as being data valid for 2005.

**Table 4:** Predictors of the number of mammography units in 27 countries, from a least square regression model^a^ including all variables in table

**Variable**	**Beta coefficient**	**95% confidence interval**	***P*-value**
Number of radiologists	0.26	0.08; 0.45	0.0081
Total female population (in million)	0.35	0.04; 0.67	0.035
Country surface (in thousand square kilometer)	0.09	−0.01; 0.18	0.077
Constant	−447	−713; −180	0.0029

a *R*^2^ of model=0.86.

## References

[bib1] Advisory Committee on Breast Cancer Screening (ACBCS (2006) Screening for breast cancer in England: past and future. NHSBSP Publication No 61. February 2006 http://www. cancerscreening.nhs.uk

[bib2] Brown ML, Kessler LG, Rueter FG (1990) Is the supply of mammography machines outstripping need and demand? Ann Intern Med 113: 547–552 2393209 10.7326/0003-4819-113-7-547

[bib3] COCIR European Coordination Committee of the Radiological and Electromedical Industries (2003) Age Profile Medical units, third edition ‘The Need for Sustained Investment’, http://www.cocir.org (last accessed 01/04/2008)

[bib4] Colbert JA, Kaine EM, Bigby JA (2004) The age at which women begin mammographic screening. Cancer 101: 1850–1859 15386333 10.1002/cncr.20583

[bib5] Destouet JM, Bassett LW, Yaffe MJ, Butler PF, Wilcox PA (2005) The ACR's Mammography Accreditation Program: ten years of experience since MQSA. J Am Coll Radiol 7: 585–594 10.1016/j.jacr.2004.12.005 17411883

[bib6] Eurobarometer (2007) Health in the European Union, Special Eurobarometer 272e/Wave 66.2 Report of September 2007. Available at: http://ec.europa.eu/health/ph_publication/eb_health_en.pdf Last accessed: November 9 2007

[bib7] European Association of Radiology (EAR) (2005) Radiological Training Programmes in Europe ERA Education Survey, Analysis of Results, 2005, http://www.ear-online.org (last accessed 01/04/2008)

[bib8] Fischer R, Houn F, Van De Griek A, Tucker SA, Meyers D, Murphy M, Unis G (1998) The impact of the mammography quality standards act on the availability of mammography facilities. Prev Med 27: 697–701 9808801 10.1006/pmed.1998.0347

[bib9] Héry C, Ferlay J, Boniol M, Autier P (2008a) Trends in breast cancer incidence and mortality in middle-aged and elderly women in 28 countries with Caucasian-majority populations. Ann Oncol 19: 1009–1018 18296422 10.1093/annonc/mdm593

[bib10] Héry C, Ferlay J, Boniol M, Autier P (2008b) Quantification of changes in breast cancer incidence and mortality since 1990 in 35 countries with Caucasian-majority populations. Ann Oncol 19(6): 1187–1194 18325921 10.1093/annonc/mdn025

[bib11] Hofvind S, Geller B, Vacek PM, Thoresen S, Skaane P (2007) Using the European guidelines to evaluate the Norwegian Breast Cancer Screening Program. Eur J Epidemiol 22: 447–455 17594526 10.1007/s10654-007-9137-y

[bib12] IARC (2002) Breast Cancer Screening. IARC Handbooks of Cancer Prevention, Vol. 7, IARC Press: Lyon

[bib13] Jensen A, Olsen AH, von Euler-Chelpin M, Helle Njor S, Vejborg I, Lynge E (2004) Do non attenders in mammography screening programmes seek mammography elsewhere? Int J Cancer 113: 464–470 10.1002/ijc.20604 15455383

[bib14] Klug SJ, Hetzer M, Blettner M (2005) Screening for breast and cervical cancer in a large German city: participation, motivation and knowledge of risk factors. Eur J Public Health 15: 70–77 15788807 10.1093/eurpub/cki118

[bib15] Lynge E, Olsen AH, Fracheboud J, Patnick J (2003) Reporting of performance indicators of mammography screening in Europe. Eur J Cancer Prev 12: 213–222 12771560 10.1097/00008469-200306000-00008

[bib16] Moss SM, Cuckle H, Evans A, Johns L, Waller M, Bobrow L, Trial Management Group (2006) Effect of mammographic screening from age 40 years on breast cancer mortality at 10 years' follow-up: a randomized controlled trial. Lancet 368: 2053–2060 17161727 10.1016/S0140-6736(06)69834-6

[bib17] Organization for Economic Co-operation and Development (2007) OECD Health Data. http://www.ecosante.org (last accessed on 01/04/2008)

[bib18] Otto SJ, Fracheboud J, Looman CW, Broeders MJ, Boer R, Hendriks JH, Verbeek AL, de Koning HJ, National Evaluation Team for Breast Cancer Screening (2003) Initiation of population-based mammography screening in Dutch municipalities and effect on breast-cancer mortality: a systematic review. Lancet 361: 1411–1417 12727393 10.1016/S0140-6736(03)13132-7

[bib19] Perry N, Broeders M, de Wolf C, Törnberg S, Holland R, von Karsa L (eds) (2006) European Guidelines for quality assurance in breast cancer screening and diagnosis, 4th edn, Office for Official Publications of the European Communities: Luxembourg 10.1093/annonc/mdm481 18024988

[bib20] Population Division of the Department of Economic and Social Affairs (ESA) of the United Nations Secretariat, World Population Prospects (2007) The 2006 Revision and World Urbanization. Prospects: The 2005 Revision. Available at: http://esa.un.org/unpp/index.asp?panel=2 (Last accessed 02/10/2007)

[bib21] Smith-Bindman R, Chu P, Miglioretti DL, Quale C, Rosenberg RD, Cutter G, Geller B, Bacchetti P, Sickles EA, Kerlikowske K (2005) Physician Predictors of Mammographic Accuracy. J Natl Cancer Inst 97: 358–367 15741572 10.1093/jnci/dji060

[bib22] Smith-Bindman R, Chu PW, Miglioretti DL, Sickles EA, Blanks R, Ballard-Barbash R, Bobo JK, Lee NC, Wallis MG, Patnick J, Kerlikowske K (2003) Comparison of screening mammography in the United States and the United Kingdom. JAMA 290: 2129–2137 14570948 10.1001/jama.290.16.2129

[bib23] Spyckerelle Y, Kuntz C, Giordanella JP, Ancelle-Park R (2002) Pratiques de la mammographie chez les femmes de 35 à 75 ans : étude descriptive dans la population consultant les centres d'examens de santé. Bulletin du Cancer 89: 957–962 (article in French) 12495883

[bib24] Théberge I, Hébert-Croteau N, Langlois A, Major D, Brisson J (2005) Volume of screening mammography and performance in the Quebec population-based Breast Cancer Screening Program. CMAJ 172: 195–199 15655240 10.1503/cmaj.1040485 PMC543982

[bib25] US Preventive Services Task Force (USPSTF) (2008) Recommendations and Rationale: Screening for breast cancer. http://www.ahrq.gov/clinic/3rduspstf/breastcancer/brcanrr.htm (last accessed on 05/05/2008)

[bib26] Vatten LJ (2007) Mammography screening--time for evaluation? Tidsskr Nor Laegeforen 127(11): 1492 [Article in Norwegian] 17551550

[bib27] Voyvoda N, Ozdemir A, Gultekin S (2007) Mammography device use in Turkey, and quantity and quality analysis of mammography education. Diagn Interv Radiol 3: 129–133 17846986

[bib28] Yankaskas BC, Klabunde CN, Ancelle-Park R, Renner G, Wang H, Fracheboud J, Pou G, Bulliard JL, International Breast Cancer Screening Network (2004) International comparison of performance measures for screening mammography: can it be done? J Med Screening 11: 187–193 10.1258/0969141042467430 15624239

