# Determinants of the number of mammography units in 31 countries with significant mammography screening

**DOI:** 10.1038/sj.bjc.6604799

**Published:** 2008-11-25

**Authors:** P Autier, D Ait Ouakrim

**Correction to**: *British Journal of Cancer* (2008) **99,**, 1185–1190, doi: 10.1038/sj.bjc.6604657

The surname of Driss Ait Ouakrim was published incorrectly when this paper appeared in volume 99, issue 7. The author's name is given correctly, above.

